# Effects of Cognitive Behavioral Couple Therapy With Integrated Mindfulness on Mindful Attention, Depressive Symptoms, and Dyadic Adjustment in Low‐Income Couples: A Pilot Randomized Clinical Trial

**DOI:** 10.1111/jmft.70169

**Published:** 2026-08-31

**Authors:** Ricardo S. S. Durães, Zila Elizabeth Pulache Vargas, Ana Cristina Garcia Dias, Francisco Lotufo‐Neto, Renato T. Ramos, Antonio de Pádua Serafim

**Affiliations:** ^1^ Health Psychology Program Methodist University of Sao Paulo São Bernardo do Campo São Paulo Brazil; ^2^ Department of Social Psychology and Work University of Brasília Brasília Federal District Brazil; ^3^ Institute of Psychology Federal University of Rio Grande do Sul Porto Alegre Rio Grande do Sul Brazil; ^4^ Institute of Psychology University of Sao Paulo São Paulo São Paulo Brazil; ^5^ Department of Psychiatry Sunnybrook Health Sciences Centre, University of Toronto Toronto Ontario Canada

**Keywords:** Cognitive Behavioral Couple Therapy, depressive symptoms, dyadic adjustment, mindful attention, mindfulness

## Abstract

Psychosocial distress can exacerbate marital conflict, maladjustment, and mental health vulnerability. This pilot randomized clinical trial examined cognitive‐behavioral couple therapy (CBCT) integrated with mindfulness in low‐income Brazilian couples (per‐capita household income up to one minimum wage). Thirty‐four participants (17 heterosexual couples) were randomized (independent computer‐generated sequence) to an experimental (*n* = 16) or waitlist control group (*n* = 18). We assessed dyadic adjustment, mindful attention, marital social skills, and depressive symptoms (R‐DAS, MAAS, IHSC, BDI‐II) at baseline, post‐treatment, and 3‐month follow‐up. The intervention was eight 80‐min conjoint sessions plus daily home exercises. Time × group effects favored the experimental group for dyadic adjustment, mindful attention, and depressive symptoms (all *p* < 0.001, $\eta_{p}^{2}$ = 0.20–0.37), but not marital social skills (*p* = 0.14). Because two outcomes differed at baseline, effects were confirmed with baseline‐ and dependence‐adjusted sensitivity analyses. These findings provide preliminary evidence that CBCT with mindfulness may benefit disadvantaged couples.

## 1 Introduction

Marital social skills, mindful attention, and, depending on the situation, problem‐solving and communication skills are critical abilities for sustaining a healthy partnership. However, couples living under socioeconomic disadvantage often face structural and financial barriers to accessing relationship and mental health resources, including cost, uncertainty about where to seek help, and broader contextual constraints associated with low socioeconomic status, which may limit opportunities to develop these capacities (Williamson et al. 2019; Steele et al. 2007; Karney 2021). The process of solving marital problems and reducing tensions often requires specialized professional support (A. T. Beck 1989; Bodenmann et al. 2014; Dattilio 2009; Dattilio and Padesky 1990; Del Prette et al. 2008). Direct interventions on relationship conflicts, sometimes combined with meditation, have proved to be particularly beneficial to promote health and potentially prevent mental and physical illness (Carson et al. 2004; Robles et al. 2014). These structural realities make the everyday material context of low‐income couples an essential consideration for any relationship intervention.

Living at or below one minimum wage per capita carries substantial material and health consequences in Brazil. During this study, the nominal minimum wage represented only about one‐fifth of the income Dieese estimated a four‐person family needed for basic subsistence (5.27 times the minimum wage; Departamento Intersindical de Estatística e Estudos Socioeconômicos 2021). Income distribution is the principal determinant of food insecurity in the country (Jesus et al. 2024): according to national survey data, roughly 72% of households experiencing moderate‐to‐severe food insecurity live on an income of up to one minimum wage per capita (Instituto Brasileiro de Geografia e Estatística 2024). Such chronic material strain compounds the stress, depressive symptoms, and relationship instability that are more prevalent among socioeconomically disadvantaged couples (Karney 2021; Hogendoorn et al. 2022; Dew et al. 2012), reinforcing the relevance of accessible relational and mental‐health interventions for this population.

## 2 Mental Health and Well‐Being in Marital Functioning

Although satisfaction in romantic relationships is subjective and multifaceted, it plays a central role in dyadic adjustment and requires continuous adaptation, particularly in the daily lives of low‐income couples. In addition to couple‐specific dynamics, this perception is shaped by psychological, cognitive, and emotional processes, as well as by cultural, social, and family characteristics (A. T. Beck 1989; Dattilio 2009; Dattilio and Padesky 1990; Mosmann and Falcke 2011). In this context, adverse relational experiences may increase vulnerability to psychiatric disorders, especially depression (Beach 2014; Neves and Duarte 2015; Kronmüller et al. 2011; Robles 2014), and are also associated with poorer immune functioning (Kiecolt‐Glaser 2018) and cardiovascular health (Robles et al. 2014; Sbarra et al. 2015; Wong et al. 2018). Among socioeconomically disadvantaged populations, chronic financial strain and lower educational attainment have been linked to greater relationship distress and a higher risk of union dissolution (Karney 2021; Hogendoorn et al. 2022; Dew et al. 2012). Depressive symptoms may also be more common among married individuals with lower educational levels (Choi and Marks 2013), although vulnerability and resilience in the face of marital conflict and divorce vary considerably (Perrig‐Chiello et al. 2015). Chronic stress may further intensify emotional distress through physiological pathways, including immune dysregulation, sustained cortisol activation, and reduced health and quality of life (Basharpoor and Sheykholeslami 2015; Vilela 2010; Shrout et al. 2020). Communication and self‐monitoring skills may function as protective factors for marital satisfaction and reduced depression (Babaee and Ghahari 2016; Miller et al. 2013; Whisman and Kaiser 2008), whereas depressive symptoms may worsen marital satisfaction and dysfunctional communication styles (Qadir et al. 2013; Rehman et al. 2010; Cohen et al. 2010; Cohen et al. 2014). In this context, Cognitive Behavioral Couple Therapy may represent a valuable intervention for low‐income couples. Taken together, these findings suggest a pathway whereby socioeconomic disadvantage heightens chronic stress and depressive symptoms, which in turn erode communication and dyadic adjustment, underscoring the need for accessible, low‐cost relational interventions for this population.

## 3 Cognitive Behavioral Couple Therapy

Briefly, Cognitive Behavioral Couple Therapy (CBCT) seeks to identify and modify dysfunctional behaviors, distorted cognitions, and emotional responses that contribute to the onset and maintenance of marital conflict (Dattilio 2009; Dugal et al. 2018; Fischer et al. 2016). It also addresses the attributions and inferences partners make about positive and negative relationship events, as well as schemas shaped by lifelong experiences that may adversely affect the current relationship. Contemporary CBCT conceptualizes couple well‐being within a broad contextual framework that includes each partner, the dyad, and the environment in which the relationship develops (Epstein et al. 2019).

A central focus of CBCT is the restructuring of cognitions that may drive maladaptive behaviors. Therapists attend to cognitive processes involved in relationship dysfunction, including selective attention, cognitive distortions such as all‐or‐nothing thinking and mind reading, and related cognitive factors. These include attributions about marital events, assumptions regarding marriage, unrealistic expectations about the likelihood of relationship events, and patterns of belief concerning the characteristics a partner should have (Baucom et al. 2015; Christensen et al. 2004; Dattilio 2009; Durães et al. 2020).

CBCT sessions are also educational in nature, aiming to engage participants actively in the therapeutic process (Dattilio 2009; Dattilio and Padesky 1990; Dugal et al. 2018; J. S. Beck 2011). Another important emphasis is the role of negative automatic thoughts in shaping cognitive, emotional, and behavioral reactions that may increase vulnerability to conflict in romantic relationships (A. T. Beck 1989; Dattilio 2009; Leahy 2003). Accordingly, CBCT targets partners’ interpretations of relationship events to promote more adaptive appraisals, reduce reactivity, and improve interaction patterns (Dattilio 2009; Baucom et al. 2015; Epstein et al. 2019; Rector and Cassin 2010). In sum, CBCT provides a structured, couple‐focused framework for reducing conflict and reactivity, offering a natural basis for integrating mindfulness.

## 4 Mindfulness in Interpersonal Relationships

According to Shapiro et al. (1998), mindfulness refers to a state of present‐moment awareness that may increase empathy, promote acceptance, reduce avoidance in romantic relationships (Wachs and Cordova 2007), strengthen social connection (Deci and Ryan 1991), improve social skills and perception (Schutte et al. 2001), and inhibit negative reactivity during conflict (Baer 2003). Mindfulness‐based practices have also been associated with better quality of life, greater marital satisfaction, and more positive affect. In this sense, mindfulness involves purposefully paying attention to internal and external experiences with openness and without judgment (Kabat‐Zinn 2003; O'Leary et al. 2016).

Because distractions and task‐unrelated thoughts about past or future events may intensify suffering (Germer 2013), mindfulness practice cultivates attentive awareness of the present moment while reducing avoidance of emotional experience (Dattilio 2009; Kabat‐Zinn 2013). A mindful stance is linked to attentional self‐regulation, emotional alertness, flexible self‐assessment, resilience, and the identification and prevention of behavioral avoidance, thereby creating conditions for more functional emotional responses and greater empathy (Dattilio 2009; Germer 2013; J. S. Beck 2011). Mindfulness may therefore influence couple functioning by enhancing present‐moment attention and emotion regulation, which shape how partners perceive and respond to relational stress and conflict (Tang et al. 2015).

Within CBCT, mindfulness is conceptualized as a learnable skill that may reduce reactivity to positive, negative, or neutral events and improve marital harmony, closeness, and satisfaction (Dattilio 2009). Empirical studies and reviews suggest positive associations with relationship satisfaction, emotional skills, attentive listening, empathic communication, and dyadic stress reduction (Wachs and Cordova 2007; Barnes et al. 2007; Kabat‐Zinn 2013; O'Kelly and Collard 2012; Winter et al. 2021; Voldstad et al. 2025). However, most mindfulness‐based interventions have focused on enhancing well‐being in non‐distressed couples rather than integrating mindfulness with established treatments such as CBCT for socially and financially vulnerable populations.

Several mindfulness‐informed approaches to couples already exist, yet none targets the population and format addressed here. Mindfulness‐Based Relationship Enhancement (Carson et al. 2004) was designed to enrich non‐distressed, mostly middle‐class couples rather than to treat conflict. Mindfulness‐Based Cognitive Therapy and MBSR (Kabat‐Zinn 2013; Segal and Walsh 2016) are individual protocols targeting depressive relapse and stress, not dyadic functioning. Acceptance‐oriented couple models such as Integrative Behavioral Couple Therapy (Christensen et al. 2004) incorporate acceptance as a principle but do not deliver a standardized, formal mindfulness curriculum. For the same reason, we integrated mindfulness into CBCT rather than adopting Acceptance and Commitment Therapy (ACT): although ACT incorporates mindfulness, it is an individually oriented model centered on psychological flexibility and does not provide the dyadic cognitive restructuring and marital social‐skills training central to managing couple conflict. CBCT offers a couple‐specific structure with established evidence for relationship distress (Fischer et al. 2016), to which mindfulness adds attentional and emotion‐regulation capacities that buffer reactivity during conflict. Against this background, the present protocol is distinctive in integrating structured formal and informal mindfulness into an eight‐session CBCT protocol, in targeting socioeconomically disadvantaged, low‐education couples (90% Black or Mixed‐race in the present sample) largely absent from the predominantly WEIRD evidence base (Foale et al. 2024), and in relying on accessible technology and a hybrid in‐person/remote format.

## 5 Present Study

The present study aimed to evaluate the effectiveness of an integrated CBCT and mindfulness‐based protocol for couples from low socioeconomic backgrounds. Specifically, we examined its effects on dyadic adjustment, mindful attention, depressive symptoms, and marital social skills.

We tested two hypotheses, organized by outcome level. *H*_1_: At the relational level, the integrated CBCT–mindfulness protocol would improve dyadic adjustment and marital social skills; *H*_2_: At the individual level, it would increase mindful attention and reduce depressive symptoms in each partner.

This is a pilot proof‐of‐concept study aiming to develop treatment tools accessible to public health intervention teams working with low‐income populations. In addition to promoting healthy marital relationships and reducing the risk of psychiatric morbidities, the development of effective couple interventions has the potential to reduce the incidence of psychological and physical abuse between partners and prevent domestic violence. Although the present trial did not assess intimate partner violence outcomes, improving dyadic communication and reducing relational distress may be relevant to broader prevention efforts in contexts marked by gender‐based violence. This study also addresses a critical gap in the literature identified by recent works (e.g., Foale et al. 2024; Le et al. 2025; Tian et al. 2025) regarding the scarcity of mindfulness research involving low‐income and less educated couples. This study was designed as a first step toward validating a simplified CBCT‐Mindfulness protocol, including in‐person and virtual appointments, that hopefully can contribute to the democratization of evidence‐based relational health interventions.

## 6 Methods

This study was designed as a pilot randomized clinical trial. Participants were low‐income heterosexual couples allocated either to an experimental group receiving the integrated CBCT‐mindfulness intervention or to a waitlist control group.

### 6.1 Participants

The final sample comprised 34 Brazilian participants (17 heterosexual couples, married or cohabiting). Of these, 16 participants (eight couples) were allocated to the experimental group (EG) and 18 participants (nine couples) to the control group (CG). Most participants were between 31 and 40 years old (*n* = 19, 55.9%). Regarding educational level, having completed high school was the most frequent category (*n* = 20, 58.8%). In terms of occupation, 19 participants (55.9%) reported being employed. Concerning family income, most couples reported a monthly family income of up to BRL 2000.00 (*n* = 9, 52.9%), followed by BRL 2001.00 to 4000.00 (*n* = 5, 29.4%) and BRL 4001.00 to 5600.00 (*n* = 3, 17.6%). With regard to children, 12 participants (35.3%) reported having one child. Regarding relationship length, seven couples (41.2%) had been together for 1–7 years, and another seven couples (41.2%) for 8–15 years. Finally, 26 participants (76.5%) reported attending weekly religious services, and 30 (88.2%) were in their first marriage. Ninety percent of the sample reported as being Black or Mixed people, and 10% White (details in Table 1).

**Table 1:** Sociodemographic characteristics of the sample.

	EG (*N* = 16)		CG (*N* = 18)		Total (*N* = 34)	
	*n*	%	*n*	%	*n*	%
Age						
18–20 (years)	—	—	1	5.5	1	2.9
21–30 (years)	5	31.2	5	27.8	10	29.4
31–40 (years)	9	56.2	10	55.5	19	55.9
41–50 (years)	2	12.5	1	5.5	3	8.8
≥ 50 (years)	—	—	1	5.5	1	2.9
Educational level						
Elementary school	3	18.7	5	27.8	8	23.5
Incomplete high school	1	6.25	1	5.5	2	5.9
Complete high school	10	62.5	10	55.5	20	58.8
Incomplete higher education	2	12.5	2	11.1	4	11.8
Occupation						
Employee	9	56.2	10	55.5	19	55.9
Unemployed	3	18.7	—	—	3	8.8
Self‐employed	1	6.25	4	22.2	5	14.7
Out of the workforce^b^	3	18.7	4	22.2	7	20.6
Monthly family income^a^						
Up to R$ 2000.00	4	50	5	55.6	9	52.9
R$ 2001.00–4000.00	3	37.5	2	22.2	5	29.4
R$ 4001.00–R$ 5600.00	1	12.5	2	22.2	3	17.6
Children						
None	2	12.5	6	33.3	8	23.5
One	6	37.5	6	33.3	12	35.3
Two	6	37.5	3	16.7	9	26.5
Three or more	2	12.5	3	16.7	5	14.7
Length of marriage^a^						
1–7 (years)	4	50	3	33.3	7	41.2
8–15 (years)	2	25	5	55.6	7	41.2
≥ 16 (years)	2	25	1	11.1	3	17.6
Weekly religious service						
No	3	18.7	5	27.8	8	23.5
Yes	13	81.2	13	72.2	26	76.5

Abbreviations: CG, control group; EG, experimental group.

^a^ Based on a couple.

^b^ Neither works nor looks for.

A statistical power analysis was performed for sample size estimation, based on G*Power 3.1.9.7. The A priori analysis results for ANOVA‐Repeated mixed measures were Effect size *f* = 0.30 (medium), with an alpha (*α* err prob) = 0.05 and power (1 − *β* err prob) = 0.80, number of groups = 2, and measurements = 4. The projected sample size needed with this effect size was *N* = 18. Thus, our final sample size was adequate for the main objective of this study. No participants received any compensation for participating in this study.

### 6.2 Inclusion and Exclusion Criteria

**Figure 1:**
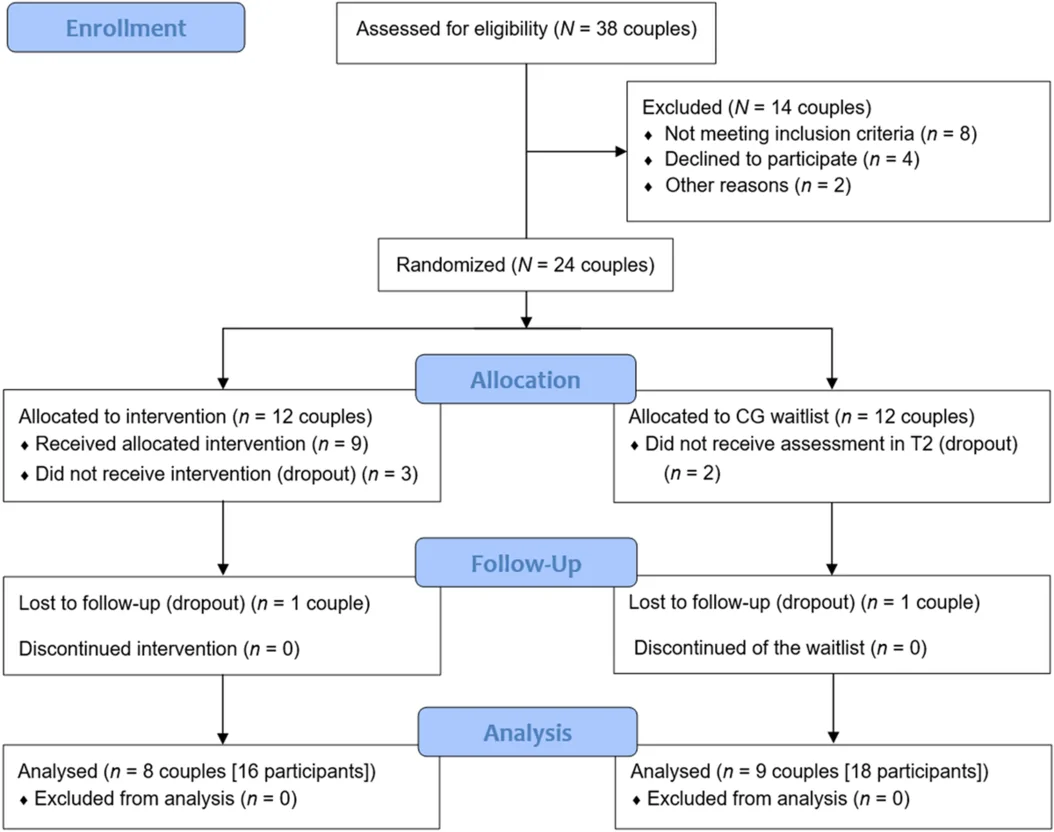
Flow diagram of the study. [Color figure can be viewed at wileyonlinelibrary.com]

The inclusion criteria were as follows: age between 18 and 60 years; being in a heterosexual relationship; Portuguese as the native language; literacy; and educational attainment up to completed high school, or current enrollment in the first semester of higher education; having one or more year of marriage or cohabitation; to present conflict in the relationship related to communication difficulties (we used the Romantic Partner Conflict Scale adapted); no regular prior mindfulness or meditation practice (operationalized as no formal practice within the previous 6 months, verified by self‐report during the initial screening interview); monthly family income up to BRL 5600.00 (Brazilian reais) (approximately five times the Brazilian minimum wage), a threshold close to the Dieese (2021) estimate of the monthly income required to meet the basic needs of a four‐person household in Brazil during the study period; having consent of both spouses to participate in the study. Exclusion criteria were current psychotic symptoms, acute suicide risk, current alcohol or other substance dependence, use of psychiatric medication requiring clinical monitoring, severe cognitive impairment interfering with participation, or any acute psychiatric condition requiring immediate specialized care. All participants included in this study filled out and signed the Informed Consent Form (ICF) approved by the research ethics board. The trial followed the CONSORT (CONsolidated Standards Of Reporting Trials) reporting guidelines (Figure 1).

Low income was operationalized using a per‐capita household income criterion expressed in multiples of the national minimum wage, consistent with the socioeconomic stratification commonly applied in Brazilian research, which ranks households into classes A–E, from highest to lowest, on the basis of per‐capita household income (Neri 2024). In this framework, the lowest stratum (class E) corresponds to a per‐capita monthly income of up to one minimum wage. Eligibility was therefore set at a total monthly family income of up to BRL 5600 (approximately five times the minimum wage during this study), a threshold close to the Dieese (2021) estimate of the income required to meet the basic needs of a four‐person household. Given the household sizes and incomes reported, the majority of participants fell in the lowest income stratum (class E), with a per‐capita income below one minimum wage.

### 6.3 Measures

We assessed four constructs: dyadic adjustment (the couple's consensus, satisfaction, and cohesion), mindful attention (the disposition to attend to present‐moment experience), marital social skills (assertive, expressive, and self‐regulatory behaviors in the relationship), and depressive symptoms (the severity of self‐reported depressive affect and cognition), each operationalized by a validated instrument described below.

#### 6.3.1 Dyadic Adjustment

The dyadic adjustment was assessed with the Revised‐Dyadic Adjustment Scale (R‐DAS; Busby et al. 1995; Portuguese version validated by Hollist et al. 2012). The scale is composed of 14‐items which seek to represent marital adjustment through three dimensions: dyadic consensus, dyadic satisfaction, dyadic cohesion. The instrument is answered using a Likert‐type scale ranging from 0 to 5 to 0–4 points, generally meaning between “*we always agree*” and “*we never disagree*,” “*always*” and “*never,*” “*every day*” and “*never*,” and “*more than once a day*.” The total score of the scale can range from 0 to 69 points. How much the higher the score, the greater the adjustment. In the current study, Cronbach's alphas for R‐DAS ranged from 0.75 to 0.80 for EG across three times of measurement (average *α* = 0.77), and from 0.74 to 0.83 for CG (average α = 0.78). The overall internal consistency was good (*α* = 0.78).

#### 6.3.2 Mindful Attention

The mindful attention's perception was measured with Mindful Attention Awareness Scale (MAAS; Brown and Ryan 2003; Portuguese version validated by Barros et al. 2015) The scale is a 15‐item Likert‐type scale (6 = *almost never*; 1 = *almost always*), with the aim of evaluating attention focused on the consciousness of the moment present. Respondents should indicate how much they have experienced what is described in each of the statements, using a 6‐point Likert scale, ranging from 1 (*almost always*) to 6 (*almost never*). Cronbach's alpha for the Brazilian adaptation was *α* = 0.83. In the current study, the internal consistencies for MAAS ranged from 0.80 to 0.87 for EG across three times (average *α* = 0.84), and for CG from 0.75 to 0.88 (average *α* = 0.83). Overall Cronbach's alpha had good internal consistency (*α* = 0.83).

#### 6.3.3 Marital Social Skills

Marital social skills quality was measured with the Marital Social Skills Inventory (IHSC; Villa and Del Prette 2012). It is a 32‐item Brazilian instrument. The instrument evaluates assertive communication, expression of positive feelings/pleasure, expression of disagreement/displeasure, disinhibition/spontaneity, and aggressiveness control. The scale Likert ranges from 1 (*never or rarely*) to 5 (*always* or *almost always*). Meaning of the score: The higher the score, the more marital social skills. In the current study, Cronbach's alphas ranged from 0.81 to 0.90 for EG across three times of measurement (average *α* = 0.86), and from 0.82 to 0.87 for CG (average *α* = 0.84). Overall global for IHSC had good internal consistency (*α* = 0.85).

#### 6.3.4 Depression Symptoms

Depressive symptoms were assessed with the Beck Depression Inventory (BDI‐II; Beck et al. 1996; Portuguese version validated by Cunha 2001). The 21‐item self‐administered instrument aims to measure the intensity of depression from age 13 through old age. There is no time limit for completing the protocol, but, in general, the BDI‐II requires between 5 and 10 min to complete. Each response is assigned a value from 0 to 3. Evaluates from minimal symptoms to severe ones. In this study, Cronbach's alphas ranged from 0.80 to 0.91 for EG across three times of measurement (average *α* = 0.84), and from 0.84 to 0.90 for CG (average *α* = 0.87). The overall internal consistency was good (*α* = 0.86).

### 6.4 Procedures

#### 6.4.1 Ethics Approval

This study was approved by the National Council of Ethics in Research (CONEP) of the Methodist University of São Paulo‐Brazil (Approval: CAAE 09911419.1.0000.5508, ID 3.257.453). The written Informed Consent Term (ICF) was obtained from all participants before the study. The study was registered in the Brazilian Clinical Trial Register under number RBR‐66n5c5r.

#### 6.4.2 Recruitment

The volunteers were recruited by disseminating study information in schools, universities, and churches, through posters and oral presentation. Those interested contacted those responsible for the research by phone, WhatsApp, or email and, from then on, went through the registration and selection processes. In order to verify the presence of conflicts in the marital relationship for inclusion in this study, we used 12‐questions adapted from the Romantic Partner Conflict Scale (Zacchilli et al. 2009). Items from two factors were used: 6 from interactional reactivity (verbal aggression and emotional response) and 6 from Domination (trying to take control in a conflict situation).

#### 6.4.3 Enrollment and Screening

When contacting the research team, volunteers underwent an initial screening, carried out remotely (phone and/or internet), and received detailed information about the research, such as inclusion criteria, research stages, and evaluation forms, including that they could be part of an intervention group or a waiting list. Those who met the criteria to enter the second phase of the screening were referred to the following week to attend in person, in order to assess their mental health, thus filling out the General Health Questionnaire (QSG‐12). The relationship with the use of alcohol was also evaluated, thus completing the Screening Test for Involvement with Alcohol, Cigarettes and Other Substances—ASSIST (Alcohol, Smoking, and Substance Involvement Screening Test and interview with the couple to identify the real interest both in participating in the research.

#### 6.4.4 Randomization

All participants who went through the screening and enrollment processes were allocated into two groups (experimental group—EG, which underwent intervention, and a waitlist control group—CG). The allocation sequence was generated by an independent assistant using a computer‐based randomization software procedure (randomizer.org/). Participant enrollment was conducted by the study research assistants, while the assignment of participants to their respective groups was performed by research coordinators who were not involved in the outcome assessment.

Couples were enrolled on a rolling basis as they expressed interest and met eligibility, and randomized upon completing the baseline assessment. Because therapists delivered the protocol across successive couples, increasing therapist familiarity over time cannot be ruled out and is acknowledged as a limitation.

#### 6.4.5 Blinding

Due to the nature of the intervention, participants and therapists were not blinded to group allocation. As all outcomes were assessed through self‐report measures, outcome assessment was also not blinded. However, the allocation sequence was generated independently, and participant assignment was conducted by researchers who were not involved in treatment delivery. Data analysis was performed under blinded conditions, with groups coded before the analytical procedures were conducted.

#### 6.4.6 Intervention

Before being allocated for any of the two groups, participants were assessed at T1‐ pre‐intervention (assessments were conducted at baseline), and then EG couples received intervention (8 weeks), and the CG were assigned to waiting list. EG Couples received eight conjoint 80‐min therapy sessions distributed over an 8‐week period in accordance with the protocol tested in this study. The sessions occurred weekly, except for the last two (seventh and eighth), which took place in an interval of 15days. In the week following the end of the intervention, each couple was invited to another meeting, in which they completed the measurement instruments (R‐DAS, IHSC, MAAS, and BDI‐II). The entire process, from the beginning of the intervention (EG), carried out within a period of 12 weeks for each couple. Assessments post‐treatment (T2) were immediately after the intervention. After 3 months, there was a follow‐up evaluation (T3), and the couples completed the instruments again. Throughout the intervention, couples had contact with the therapist once a week via text or voice message or via WhatsApp, in order to address issues related to homework. Each couple had approximately 10–15 min of contact per week. The entire process, from the first screening to the follow‐up assessment, lasted 28 weeks, approximately 7 months per couple. We used an app to carry out mindfulness practices and an iPad to show images and short videos related to the therapeutic process, such as training and psychoeducation sessions (Table 2).

**Table 2:** Intervention protocol of this study.

Session	Topic content	Homework
1st	Psychoeducation and instructions on Mindfulness practices.	Mindfulness^a^, book therapy.
2nd	Attributions, assumptions, patterns, expectations, and marital functioning.	Mindfulness^a^, identifying patterns, attributions, expectations, and assumptions.
3rd	Automatic thoughts, emotions, and cognitive distortions.	Mindfulness^a^, identifying and registering cognitive distortions.
4th	Marital social skills training and communication.	Mindfulness^a^, reading and assessment of communication assertiveness.
5th	Communication training and role‐playing.	Mindfulness^a^, recording of automatic thoughts during the couple's dialogues and discussions.
6th	Cognitive restructuring and change of thinking.	Mindfulness^a^, Dysfunctional Thought Record.
7th	Problem solving training; Role‐Playing.	Mindfulness^a^, problem solving and 5W2H^b^ action plan.
8th	Relapse prevention.	mindfulness^a^
**Step**	**Structure in‐session**	**Time (≈ 1 h 20 min)**
1	Mood assessment and exam/update of the week	2 min
2	Homework review	10 min
3	Mindfulness practice	10 min
4	Bridge from the previous session to the present one	5 min
5	Week update and development of the agenda of the current session (collaboration)	35 min
6	Definition of new homework	5 min
7	feedback session	10 min

^a^ 10 min of in‐conjoint meditation guide + at least 10 min for mindful walking and 10 min for mindful eating. The exercises were performed at least 5 days per week.

^b^ 5W2H = “What?” “Why?” “Who?” “Where?” “When?” “How?” and “How much does it cost (if applicable)?”

#### 6.4.7 Mindfulness Practices

In addition to the in‐session practices, participants were instructed to engage in daily home‐based mindfulness exercises using guided audio practices delivered through the Ácora app (Mindfulness Meditation, Version 1.0), available free of charge for Android and iOS. In the present study, the app functioned solely as a vehicle for delivering mindfulness audio guides, which constituted the core component of the home‐based practice. No participant‐identifiable data were collected, accessed, or monitored through the app by the research team. The authors had no commercial, financial, or institutional relationship with the app developers. The user chose the practice time and followed his own evolution, in addition to programming, as a reminder, the exercise moments. The formal guided practices lasted approximately 10‐min each, per day. The homework exercises were body scan, practiced together with the partner, and informal Mindfulness practices in order to develop mindful attention to everyday routines, such as eating and walking with attention to the action (mindful walking and mindful eating).

According to Kabat‐Zinn (2013), the guided body‐scan audio was intended to support practice carried out naturally, without effort or judgment, and to help participants sustain a regular routine. The specific practices, doses, and schedule are summarized below. Couples in the experimental group exchanged chat messages and voice/video conferencing with the therapist, once a week, in intervals between sessions, for approximately 10–15 min, in order to update each other or clarify doubts about homework.

The home‐based mindfulness protocol can be summarized as follows. Delivery: home practice was delivered via free guided‐audio recordings in the Ácora app, used solely as an audio‐delivery vehicle (no participant data collected). Formal practices: body scan (≈ 10 min/day, 5 days/week, with the partner) and back‐to‐back (with the partner) breathing, sitting meditation once a week. Informal practices: mindful walking (≈ 15 min, 3×/week) and mindful eating (3×/week), alone and with the partner. Dose and schedule: the target was 30–45 min of practice per day, with participants choosing practice times and setting app reminders to support adherence (Kabat‐Zinn 2013).

#### 6.4.8 Psychotherapists

Three therapists were involved in the screening process and provided support to the participants. All of them were licensed psychologists. All therapists participated part‐time, received training and supervision by two PhD in clinical psychology specialized in couple therapy and mindfulness‐based therapies once per week lasting approximately 1 h (initial protocol training was completed before the trial began, and weekly supervision continued throughout the intervention period to support treatment fidelity). Ratings were performed by two independent raters trained in delivering both treatments.

### 6.5 Data Analysis

**Figure 2:**
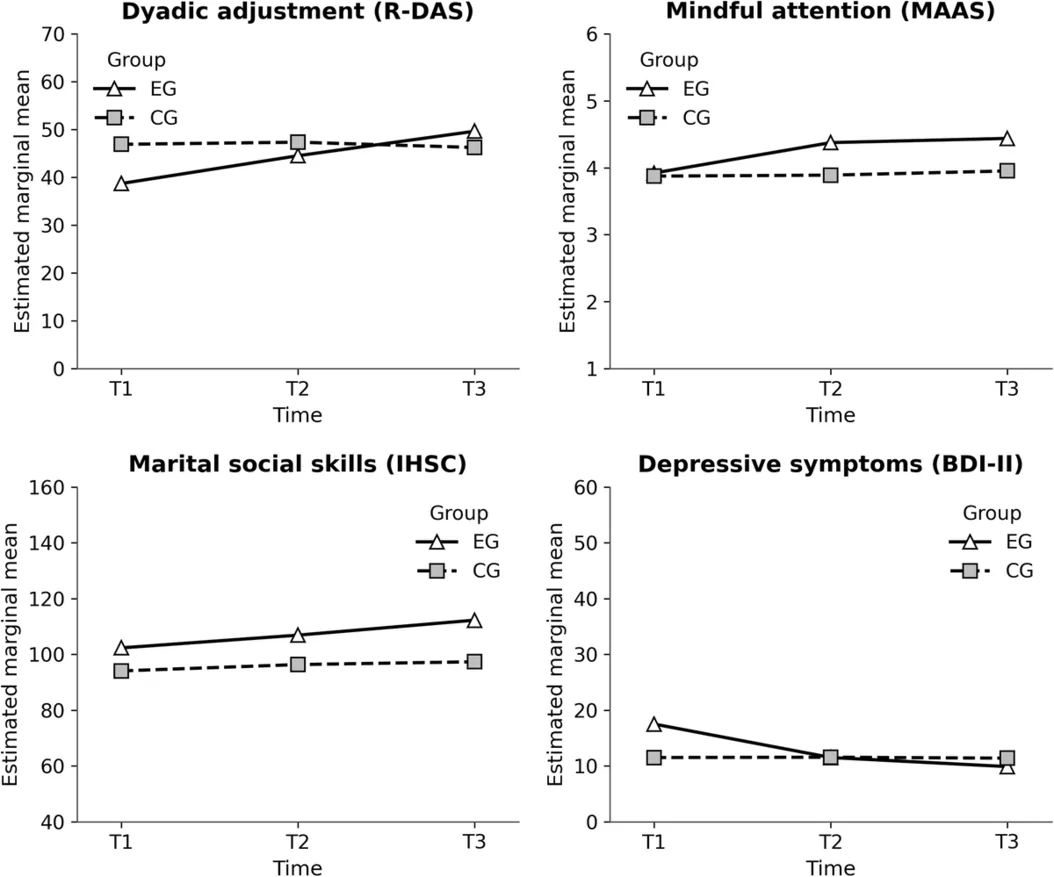
Estimated marginal means for EG and CG in the three times. The plotted values are the unadjusted estimated marginal means of the repeated measures mixed ANOVA, that is, the observed trajectory of each group. The baseline‐adjusted comparisons are reported in the Baseline equivalence subsection of the Results.

**Figure 3:**
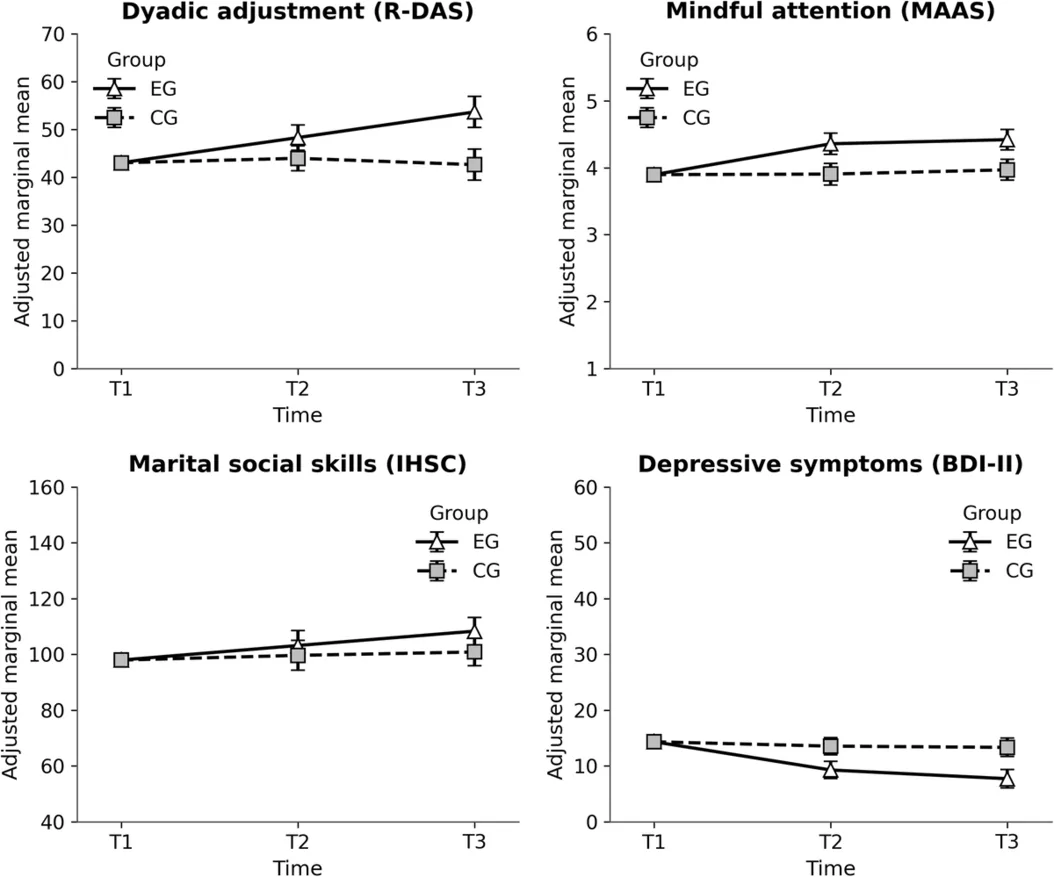
Baseline‐adjusted estimated marginal means for EG and CG. *Note:* Values are adjusted means from the analyses of covariance described in the Data Analysis section, with the baseline score of each outcome as covariate and a random intercept for couple; both groups are therefore equated at T1 at the sample baseline mean. Error bars are 95% confidence intervals for the adjusted between‐group difference. Observed (unadjusted) means are presented in Figure 2 and Table 3.

Data were analyzed using IBM SPSS Statistics for Windows, version 23.0 (IBM Corp., Armonk, NY, USA), and Figures 2 and 3 were generated in Python 3.12 (Python Software Foundation, Wilmington, DE, USA) using Matplotlib 3.10 (Matplotlib Development Team, NumFOCUS, Austin, TX, USA). Data analysis of this randomized clinical trial with two groups, one treatment and one control, was performed using a Mixed Analysis of Variance (ANOVA) (Repeated Measures Mixed ANOVA) with two factors and repeated measures, considering time (pre, post‐intervention, and follow‐up) as a within‐individual/group factor and groups as a between‐individual/group factor. Analyzes showed the interaction effect of this ANOVA. For all analyses, *p*‐value ≤ 0.05 was considered significant.

Baseline equivalence between groups was tested for all sociodemographic variables (chi‐square [$x^{2}$] or Fisher's exact tests at the couple level) and for all outcomes (independent‐samples *t*‐tests with Levene's test for equality of variances). Because the groups differed at baseline on dyadic adjustment and depressive symptoms, the mixed ANOVAs were supplemented with analyses of covariance (ANCOVA) in which the follow‐up score of each outcome was the dependent variable, group was the fixed factor, and the baseline score of that same outcome was the covariate; because partners are nested within couples, these models also included a random intercept for couple (Vickers and Altman 2001). This model estimates the difference between groups at follow‐up once both groups are equated on their pre‐treatment scores, so that a significant group term indicates a difference that is not explained by baseline standing alone. Homogeneity of variance was not satisfied for dyadic adjustment or marital social skills; mixed models and Welch‐corrected tests, which do not assume equal variances, were used accordingly. Because partners are nested within couples, the two members of each dyad are not independent; we therefore conducted two sensitivity analyses, refitting each outcome with a linear mixed model including a random intercept for couple (participants nested within couples) and testing the time × group interaction via likelihood‐ratio tests, and repeating the time × group analyses separately for men and women.

All outcomes, the individual‐level constructs (mindful attention and depressive symptoms) and the relational constructs (dyadic adjustment and marital social skills), were analyzed at the level of the individual participant.

Two effects of the mixed ANOVA are reported for every outcome, and they answer different questions. The time × group interaction tests whether the two groups changed differently across the three assessments and is therefore the effect of interest for intervention efficacy. The between‐subjects effect tests whether the groups differ in the mean of their scores across the three assessments, that is, in their overall level, regardless of the shape of change. Because the experimental group began from a more distressed position and then improved, its lower pre‐treatment scores and higher follow‐up scores tend to offset each other in that mean; a significant interaction combined with a non‐significant between‐subjects effect is therefore expected here and does not contradict the conclusion that the groups changed differently over time.

The Post Hoc Test was used for multiple comparisons of means observed in equal variances assumed. Descriptive statistics, effect size estimates, homogeneity tests, and Bonferroni‐adjusted confidence intervals were also used, with a significance level of 0.05 and a 95% confidence interval. The homogeneity test checked the variances for each group combination of its two factors. The studentized (i.e., internally standardized) residuals checked that there were no significant outliers in any within‐individual/group or between‐individuals/groups factor group in order to determine that the dependent variable was distributed approximately normally for each combination of its factor groups.

The variances of differences between all combinations of within‐subject/group factor groups were checked by Mauchly's sphericity test. The magnitude of effect size and interaction was presented by partial eta square ($\eta_{p}^{2}$) and interpreted as weak (0.01), moderate (0.06), or strong (≥ 0.14) (Cohen 1988; Dancey and Reidy 2011). The interpretation of the interactions was presented through graphs in three times T1 (pre), T2 (post), and T3 (3 months follow‐up) in order to facilitate understanding, demonstrated by estimated marginal means.

## 7 Results

Table 1 shows sociodemographic characteristics of the sample per group (EG and CG) and total.

### 7.1 Baseline Equivalence

Groups were equivalent at baseline on all sociodemographic variables at the couple level (children, *p* = 0.55; length of relationship, *p* = 0.55; family income, *p* = 1.00; age, *p* = 0.37); parental status, contrasted as childless versus parent couples, was also balanced (one childless couple in the experimental group vs. three in the control group; Fisher's exact *p* = 0.58). On the outcomes, groups were equivalent for mindful attention (*p* = 0.87) and marital social skills means (*p* = 0.38), but differed for dyadic adjustment (experimental group lower: 38.7 vs. 46.9; *p* = 0.017, *d* = −0.85) and depressive symptoms (experimental group higher: 17.5 vs. 11.5; *p* = 0.014, *d* = +0.89); variances were unequal for dyadic adjustment (Levene *p* = 0.012) and marital social skills (Levene *p* < 0.001). After adjusting for baseline scores, the experimental group showed significantly better outcomes at follow‐up for dyadic adjustment (adjusted *β* = +11.0, *p* < 0.001), mindful attention (*β* = +6.8, *p* = 0.004), and depressive symptoms (*β* = −5.6, *p* < 0.001), and no significant difference for marital social skills (*p* = 0.14). These adjusted comparisons come from a separate ANCOVA for each outcome, with the follow‐up score as the dependent variable, group as the fixed factor, and the baseline score of the same outcome as the covariate; *β* is the adjusted difference between groups (experimental minus control) expressed in the original units of each instrument, and *p* refers to the group term of that model. Because the covariate removes the variance associated with pre‐treatment standing, the persistence of the group effect after adjustment indicates that it is not attributable to baseline differences alone. The adjusted between‐group differences at follow‐up, with 95% confidence intervals, were +11.0 [4.6, 17.5] for dyadic adjustment, +6.8 [2.1, 11.4] for mindful attention on the total‐score scale (+ 0.45 [0.14, 0.76] in item means, the metric used in Table 3 and in the figures), +7.5 [ − 2.4, 17.3] for marital social skills and −5.6 [ − 8.9, −2.3] for depressive symptoms. These adjusted means are displayed in Figure 3. Figures 2 and 3, therefore, show the same four outcomes from two complementary angles and are not alternative sets of results: Figure 2 presents the trajectories that were actually observed, including the difference between the groups before treatment, whereas Figure 3 presents those trajectories after that pre‐treatment difference has been statistically removed. Reading them together shows both what happened in the sample and what remains once the groups are placed on an equal footing at baseline.

**Table 3:** Means, standard deviations, and confidence intervals on the three times by variables.

	T1 (pre‐treatment)			T2 (post‐treatment)			T3 (follow‐up^c^)		
	*M*	SD	95% CI	*M*	SD	95% CI	*M*	SD	95% CI
Dyadic adjustment									
EG^a^	38.7	4.4	33.8–43.6	44.5	3.3	38.9–50.1	49.6	6.4	43.7–55.5
CG^b^	46.9	12.6	42.2–51.5	47.3	14.7	42.1–52.6	46.2	14.7	40.6–51.8
Mindful attention									
EG^a^	3.9	0.7	3.5–4.3	4.4	0.5	4.0–4.7	4.4	0.6	4.1–4.8
CG^b^	3.9	0.9	3.5–4.3	3.9	0.8	3.5–4.2	4	0.8	3.6–4.3
Marital social skills									
EG^a^	102.3	18.3	88.1–116.5	106.9	19.0	92.0–121.8	112.2	20.3	97.2–127.3
CG^b^	94.1	34.2	80.6–107.5	96.3	35.9	82.3–110.4	97.3	35.7	83.2–111.5
Depressive symptoms									
EG^a^	17.5	6.2	14.1–20.9	11.5	5.3	8.5–14.5	9.9	5.9	6.8‐13.0
CG^b^	11.5	7.2	8.3–14.7	11.6	6.4	8.7–14.4	11.4	6.2	8.5‐14.3

Abbreviations: BDI‐II, Beck depression inventory‐II; IHSC, Marital Social Skills Inventory; MAAS, Mindful Attention Awareness Scale; R‐DAS, Revised Dyadic Adjustment Scale.

^a^ Experimental group.

^b^ Control group waitlist.

^c^ 3‐month follow‐up.

As shown in Table 4, the repeated measures mixed ANOVA for dyadic adjustment in the within‐subjects effect (time) was significant, *F*(2, 64) = 14.885, *p* < 0.001, $\eta_{p}^{2}$ = 0.317, demonstrating a strong magnitude of the effect. For time × group, the analysis was significant, *F*(2, 64) = 18.738, *p* < 0.001, $\eta_{p}^{2}$ = 0.369, demonstrating strong magnitude of the effect as well. The between‐subjects effect, which compares the groups on the mean of the three assessments, was not significant. Thus, the groups differed in how they changed over time rather than in their overall level. The scores were EG (*M*_pre_ = 38.7, SD = 4.4; *M*_post_ = 44.5, SD = 3.3; *M*_follow‐up_ = 49.6, SD = 6.4) versus CG (*M*_pre_ = 46.9, SD = 12.6; *M*_post_ = 47.3, SD = 14.7; *M*_follow‐up_ = 46.2, SD = 14.7) (see Table 3). The estimated marginal means graphic is presented in Figure 2.

**Table 4:** Analysis of variances for within and between‐subjects effects by variables.

	SS	*df*	MS	*F*	*p*	$\eta_{p}^{2}$
*Dyadic adjustment*						
Within‐subjects						
Time	453.884	2	226.942	14.885	< 0.001	0.317
Time × Group	571.374	2	285.687	18.738	< 0.001	0.369
Error (Time)	975.782	64	15.247			
Between‐subjects						
EG × CG^a^	164.461	1	164.461	0.516	0.478	0.016
Error	10,194.294	32	318.572			
*Mindful attention*						
Within‐subjects						
Time	1.631	2	0.816	12.250	< 0.001	0.277
Time × Group	1.078	2	0.539	8.092	< 0.001	0.202
Error (Time)	4.261	64	0.067			
Between‐subjects						
EG × CG	2.804	1	2.804	1.824	0.186	0.054
Error	49.185	32	1.537			
*Marital social skills*						
Within‐subjects						
Time	739.972	2	369.986	7.803	< 0.001	0.196
Time × Group	194.012	2	97.006	2.046	0.138	0.060
Error (Time)	3034.616	64	47.416			
Between‐subjects						
EG × CG	3209.562	1	3209.562	1.331	0.257	0.040
Error	77,182.516	32	2411.954			
*Depressive symptoms*						
Within‐subjects						
Time	277.818	2	138.909	17.561	< 0.001	0.354
Time × Group	268.955	2	134.478	17.001	< 0.001	0.347
Error (Time)	506.241	64	7.910			
Between‐subjects						
EG × CG	55.425	1	55.425	0.545	0.466	0.017
Error	3256.731	32	101.773			

Abbreviations: BDI‐II, Beck depression inventory‐II; *df*, degree of freedom; IHSC, Marital Social Skills Inventory; MAAS, Mindful Attention Awareness Scale; MS, mean of square; $\eta_{p}^{2}$, partial eta squared; R‐DAS, Revised Dyadic Adjustment Scale; SS, sum of squares.

^a^ Experimental group × control group waitlist.

For mindful attention, within‐subjects effect (time) was significant, *F*(2, 64) = 12.250, *p* < 0.001, $\eta_{p}^{2}$ = 0.277, demonstrating strong magnitude of the effect. For time × group, the result was significant, *F*(2, 64) = 8.092, *p* < 0.001, $\eta_{p}^{2}$ = 0.202, presenting strong magnitude of the effect as well. The between‐subjects effect, which compares the groups on the mean of the three assessments, was not significant. Thus, the groups differed in how they changed over time rather than in their overall level (see Table 4). Table 3 shows the mean scores for EG (*M*_pre_ = 3.9, SD = 0.7; *M*_post_ = 4.4, SD = 0.5; *M*_follow‐up_ = 4.4, SD = 0.6) versus CG (*M*_pre_ = 3.9, SD = 0.9; *M*_post_ = 3.9, SD = 0.5; *M*_follow‐up_ = 4.0, SD = 0.6). Figure 2 presents the estimated marginal means graphic.

Repeated measures mixed ANOVA for marital social skills showed in Table 3 significance for within‐subjects effect (time), *F*(2, 64) = 7.803, *p* < 0.001, $\eta_{p}^{2}$ = 0.196, it was strong magnitude of the effect. For the time × group, the analysis was not significant (*p* = 0.138). The between‐subjects effect was also not significant (*p* = 0.257). The groups, therefore, differed neither in how they changed over time nor in their overall level on this outcome (see Table 4). The scores were for EG (*M*_pre_ = 102.3, SD = 18.3; *M*_post_ = 106.9, SD = 19.0; *M*_follow‐up_ = 112.2, SD = 20.3) and CG (*M*_pre_ = 94.1, SD = 34.2; *M*_post_ = 96.3, SD = 35.9; *M*_follow‐up_ = 97.3, SD = 35.7) (see Table 3). The estimated marginal means graphic is presented in Figure 2. Both groups improved on this outcome over time, and the baseline‐adjusted difference at follow‐up favored the experimental group ( = +7.5 points), but its confidence interval included zero (− 2.4 to 17.3). With 17 couples, the trial was underpowered to detect a difference of this size, so this outcome should be read as inconclusive rather than as evidence that the intervention does not affect marital social skills.

Table 4 also presents the repeated measures mixed ANOVA for depressive symptoms. Within‐subjects effect (time) was significant, *F*(2, 64) = 17.561, *p* < 0.001, $\eta_{p}^{2}$ = 0.354, demonstrating strong magnitude of the effect. For time × group, the result was significant, *F*(2, 64) = 17.001, *p* < 0.001, $\eta_{p}^{2}$ = 0.347. Therefore, demonstrating strong magnitude of the effect. The between‐subjects effect, which compares the groups on the mean of the three assessments, was not significant. Thus, the groups differed in how they changed over time rather than in their overall level. According to Table 3, the mean scores were EG (*M*_pre_ = 17.5, SD = 6.2; *M*_post_ = 11.5, SD = 5.3; *M*_follow‐up_ = 9.9, SD = 5.9) versus CG (*M*_pre_ = 11.5, SD = 7.2; *M*_post_ = 11.6, SD = 6.4; *M*_follow‐up_ = 11.4, SD = 6.2). Figure 2 presents the estimated marginal means graphic.

Sensitivity analyses supported the robustness of these findings. After modeling the strong intra‐couple dependence (baseline intra‐couple correlation up to *r* = 0.91 for dyadic adjustment) with a random couple intercept, the time × group interaction remained significant for dyadic adjustment ($\chi^{2}$[2] = 31.34, *p* < 0.001), mindful attention ($\chi^{2}$[2] = 15.03, *p* < 0.001), and depressive symptoms ($\chi^{2}$[2] = 28.98, *p* < 0.001), and non‐significant for marital social skills ($\chi^{2}$[2] = 4.21, *p* = 0.12). Analyses conducted separately for men and women showed the same pattern (Supporting Information S1: Table S1); because each of these models includes only one partner per couple, observations within them are independent. The time × group interaction was significant for dyadic adjustment (men, *F*(2, 30) = 10.98, *p* < 0.001; women, *F*(2, 30) = 8.43, *p* = 0.001), mindful attention (men, *F*(2, 30) = 4.25, *p* = 0.024; women, *F*(2, 30) = 3.51, *p* = 0.043), and depressive symptoms (men, *F*(2, 30) = 7.75, *p* = 0.002; women, *F*(2, 30) = 8.41, *p* = 0.001), and non‐significant for marital social skills (men, *F*(2, 30) = 0.46, *p* = 0.634; women, *F*(2, 30) = 2.35, *p* = 0.113). Because each sex‐specific model includes only 17 participants, these analyses are underpowered and are reported as sensitivity checks rather than as confirmatory tests.

## 8 Discussion

Overall, the results obtained in this study suggest that CBCT associated with mindfulness‐based exercises may be associated with improvements in marital adjustment, mindful attention, and depressive symptoms, but not in marital social skills.

Consistent with our hypotheses, the findings provide preliminary evidence that the intervention was associated with improvements across most measured variables over time. The observed effects for dyadic adjustment and depressive symptoms corroborate previous evidences suggesting that CBCT should be considered as a frontline intervention for relational distress and individual psychopathology (Fischer et al. 2016; Bodenmann et al. 2008). The significant reduction in depressive symptoms is particularly noteworthy given the sample's vulnerability, supporting the transdiagnostic utility of mindfulness in alleviating psychological distress within a relational context (Winter et al. 2021). Regarding marital social skills, scores improved over time in both groups, but the time × group interaction was not significant (*p* = 0.138); we therefore found no evidence that the intervention outperformed the waitlist on this outcome, and Hypothesis 1 was only partially supported. One tentative reading, to be tested in adequately powered trials, is that the behavioral translation of mindful awareness into social proficiency may follow a slower trajectory. This corroborates recent findings suggesting that while mindfulness rapidly enhances emotional regulation, a precursor to skilled communication, the consolidation of complex interactional behaviors may require sustained practice, especially in populations with limited prior exposure to therapeutic models (Karremans et al. 2020; Voldstad et al. 2025). The integrated protocol may help address this gap, and future iterations might test whether an increased dosage of role‐playing exercises consolidates behavioral gains.

These findings are particularly notable given the demographic characteristics of the sample (low income and lower educational attainment). Recent systematic reviews have highlighted a “demographic gap” in mindfulness research, which predominantly targets “WEIRD” (Western, Educated, Industrialized, Rich, Democratic) populations (Foale et al. 2024). By providing preliminary evidence of benefit in a resource‐constrained context, our results are consistent with emerging evidence that digital and simplified mindfulness interventions can be successfully adapted for diverse populations (Tian et al. 2025), potentially offering greater relative gains for those with higher baseline distress due to sociodemographic disadvantage (Le et al. 2025).

### 8.1 Mindful Attention, Dyadic Adjustment, and Marital Social Skills

Concerning the effectiveness of the intervention, the results are consistent with the hypothesis that the intervention may improve participants’ levels of conscious attention and marital adjustment. This result suggests that conscious attention patterns have an important role in marital adjustment.

The development of self‐monitoring skills seems to have had an impact on the relationship adjustment between couples. Some participants in this study, during the initial interviews, reported acting “automatically” during conflicts in the marital relationship. After the intervention, many of these couples reported that they noticed changes with regard to conflict resolution, both in themselves and in their partner. This finding is in accordance with Bouchard and Saint‐Aubin (2014) and Levy et al. (2010) observations that people with deficits in executive attention may react more dysfunctional to challenging events. In these situations, instead of seeking stability and replacing automatic processes with conscious ones, they tend to be controlled by feelings and affections, and impulsive and inconsistent when facing specific situations in interpersonal relationships, for example, marital conflicts that depend on a strategy to solve them.

Mindfulness practice involves the regulation of attention by intentionally directing awareness to present‐moment experiences, such as ongoing events or bodily sensations (Kabat‐Zinn 2003, 2013). This attentional training may support problem‐solving and decision‐making by helping individuals pause and respond less automatically during interpersonal conflict. In line with this view, research on executive attention suggests that better attentional control is associated with a greater capacity to monitor and regulate responses, particularly in situations involving competing behavioral options (Holmboe and Johnson 2005; Lin et al. 2018). Likewise, Elliott et al. (2014) found that intensive meditation training may improve executive attention. However, because the present study did not directly assess executive functioning, these mechanisms should be interpreted cautiously. More broadly, mindfulness‐based approaches emphasise awareness and acceptance as conditions that may facilitate change and adaptation, although such processes are less likely to occur when individuals feel pressured to respond in a particular way (Kabat‐Zinn 2013; Lau and McMain 2005).

A conceptual tension has been noted between the change‐oriented stance of cognitive restructuring and the non‐judgmental, acceptance‐based stance of mindfulness (Lau and McMain 2005). In the present protocol we treated these as sequential and complementary rather than contradictory: mindfulness first cultivates a non‐reactive pause and de‐identification from automatic thoughts, which in turn creates the cognitive space in which deliberate restructuring can occur (Shapiro et al. 2006; Teasdale et al. 2002), and more recent work supports this sequence: decentering has been shown to increase after both mindfulness and cognitive restructuring and to operate as a mechanism common to the two techniques (Hayes‐Skelton and Lee 2020), and current conceptual analyses place mindfulness within, rather than in opposition to, the cognitive behavioral model, arguing that the attentional and metacognitive capacities it trains support the completion of restructuring exercises (Beshai 2024), a logic shared with established hybrid models such as mindfulness‐based cognitive therapy. The philosophical compatibility of acceptance‐ and change‐based strategies nonetheless remains debated and warrants further study.

Executive control provides flexibility to direct behavior toward responses that favor long‐term well‐being and has the potential to minimize the tendency to react without reflection based on negative emotions, which is a predictor of long‐term strain in the marital relationship. Mindfulness can help reduce this reactivity, as it improves executive function and allows the person to inhibit automatic reactions and choose functional and balanced responses (Karremans et al. 2015).

We tentatively propose, as a hypothesis for future testing rather than a demonstrated mechanism, that the mechanism of “pausing” before reacting is central to the integration of CBCT and Mindfulness. While CBCT provides the cognitive structure to identify distortions, mindfulness provides the physiological regulation necessary to utilize those cognitive skills during conflict (Karremans et al. 2020; Winter et al. 2021; Ciprić et al. 2022).

Jha et al. (2007) indicate that mindfulness training can improve behavioral responses related to attention; in addition, the study pointed to the development and emergence of receptive attention skills. Although the present research has not made a comparison between experienced practitioners versus beginners, it is possible to hypothesize that the combination of Mindfulness practices combined with cognitive‐behavioral interventions can explain the strong positive effect on changing behaviors and positive perception of couples, beginners in the practice, on the relationship, as well as its maintenance.

Corroborating our findings, Silva et al. (2020) reviewed studies investigating mindfulness‐based interventions for marital relationships and the results suggest that participation in such programs increases relationship satisfaction, connection with the partner, emotional balance, empathy, communication, and reduces stress, emotional reactivity, and conflicts. An experimental study (Dei 2019) in Ghana, with the objective of verifying the effect of CBT in 50 couples, with an intervention duration of 8 weeks and session duration between 50 and 110 min, demonstrated that the effect of the intervention was effective between participants with severe marital distress.

Cultivating mindfulness practices among couples might improve empathic communication, attentive and conscious listening, and improve connection between spouses, coping with conflicts, and marital satisfaction (Kabat‐Zinn 2013; O'Kelly and Collard 2012). On the other hand, Gottman (1994), Sardinha et al. (2009), and Tavakolizadeh et al. (2015) point out that couples who experience communication problems tend to distance themselves from each other and the problem tends to increase more and more. In a meta‐analysis (McGill et al. 2016) that investigated the association between Mindfulness and marital adjustment, the results were statistically significant and indicated that when an individual is more mindful, he is more satisfied with his relationship. Despite the results of Mindfulness studies and marital adjustment showing association and positive effect, it is important to consider the type (formal or informal), frequency (days/weeks), and duration (minutes) of the practice. Also, identify whether participants are new to the practice, experienced, or regular practitioners. Although the participants in the present research were inexperienced in Mindfulness practices, the results suggest that the effect was positive, both for conscious attention and for marital functioning. This time difference can be understood for a cognitive behavioral therapy combination.

In a review (Tang et al. 2015), the results indicated that mindfulness meditation has potential for the treatment of clinical disorders and can facilitate the process of recovery, mental health, and well‐being. In a qualitative study, Gillespie et al. (2015) investigated the relational experiences of partners who completed the 8‐week Mindfulness‐Based Stress Reduction (MBSR) course. The sample consisted of 11 couples with at least 2 years of relationship duration. The results showed that several intimate partners of those who completed the MBSR perceived less reactivity, calmer, and acceptance in their partner. In addition to improving emotional balance.

In the present research, some spouses reported that the practices improved their mood and well‐being, and the connection with the partner who accompanied them in the activities. The effect of skills training on slower results can be explained by the adaptation process and the need for time for changes (Bartholomeu et al. 2008; Dattilio 2009; Kabat‐Zinn 2013). In their study, Babaee and Ghahari (2016) examined the effectiveness of communication skills training on marital intimacy and adaptation for 50 participants in 10 sessions, and the results indicated that communication skills training can affect increased intimacy and adaptation relationship, but behavioral changes tend to be perceived in the long‐term. The use of technology in training and monitoring can be a positive differential in homework, such as, for example, the audios used in this research as a guide and monitoring of practices, corroborating Mausbach et al. (2010), Ciprić et al. 2022, and Tang and Kreindler (2017), who suggest the technology as a potential for positive therapeutic results. Overall, these observations converge on a consistent pattern: cultivating present‐moment awareness appears to support more adaptive couple communication and connection.

### 8.2 Effects on Depressive Symptoms

Although a mediation analysis is needed for a more accurate assessment, our results seem to corroborate the hypothesis that the intervention could contribute to reducing depressive symptoms eventually presented by the participants. Some authors, in fact, have reported that couple therapy interventions combined with mindfulness meditation have an impact on improving mental health. For example, Carson et al. (2004), Robles et al. (2014), and Kimmes et al. (2020) showed that the effects of intervention with couples, combined with meditation practices, promote health and can prevent or improve mental and physical illnesses. In a randomized clinical trial, with 64 participants, Geschwind et al. (2012) showed that Mindfulness based on cognitive therapy increases the positive effect, as well as the individual's susceptibility to rewarding situations in daily life, and reduces depressive symptoms. Segal and Walsh (2016) investigated an eight‐session online therapy program based on mindfulness and cognitive therapy focusing on affect regulation skills, using guided sessions of mindfulness meditation and CBT. Their results showed a reduction in residual depressive symptoms, reductions in rumination and increased trait mindfulness, and improvements in adaptive emotion regulation with benefits extending over 6‐months after treatment. In summary, our study suggests that the benefits of mindfulness‐based cognitive therapy, psychoeducational processes and skills development, acceptance and adaptation reported by Cohen et al. 2010; Karremans et al. 2015; Segal and Walsh 2016; Tseng et al. 2023; and van der Velden et al. 2015, can also be observed low income/low education populations. The ability to become more aware of thoughts and feelings and think in a broader perspective, is a least partially effective to minimize depressive symptoms in different populations.

### 8.3 Clinical Implications

Although several intervention tools and protocols are available for couples in distress, the therapist's clinical management remains central to treatment success. A solid understanding of cognitive, emotional, behavioral, and contextual factors may improve case conceptualization and help tailor the intervention to each couple's specific needs. The present findings suggest that integrating guided mindfulness practices with CBCT techniques focused on communication, social skills, and problem solving may enhance interventions for couples experiencing psychological distress. In particular, dyadic adjustment, mindful attention, and depressive symptoms emerged as domains in which the intervention showed preliminary benefit, whereas marital social skills did not differentiate the groups; all four may nevertheless be useful indicators to monitor over the course of treatment. Monitoring these domains may support therapists and researchers in identifying relational distress and treatment progress more precisely. Although the present pilot study does not allow firm conclusions about long‐term preventive outcomes, the intervention may have preventive and health‐promotion potential by strengthening adaptive communication, reducing emotional distress, and supporting healthier patterns of relational functioning. Because the design did not include mediation analyses or measures of emotion regulation, impulsivity, or executive functioning, we cannot identify the mechanisms underlying the observed changes. Whether improvements in dyadic adjustment are driven by enhanced emotion regulation or reduced impulsivity remains a hypothesis for adequately powered mediation studies.

### 8.4 Limitations and Future Directions

Although this research has shown positive results, some limitations must be pointed out. First, the small sample size did not allow for the investigation of important variables such as personality traits. Second, the nature of the studied population made proper dropout analysis and long‐term follow‐ups difficult. Third, despite randomization, the two groups were not equivalent at baseline on dyadic adjustment and depressive symptoms, with the experimental group starting from a more distressed position on both. The small sample precluded propensity score matching, so baseline differences were addressed with analyses of covariance, adjusting for each outcome's baseline score. Because regression to the mean cannot be entirely ruled out, all between‐group effects reported here should be regarded as preliminary and interpreted with caution.

It would be interesting for future work to replicate our results using a more representative sample to investigate the potential for providing an opportunity to expand the analyses; allocate and distribute experimental and control groups by personality traits in a proportional manner; compare a group of intervention based and CBCT with mindfulness practices with an intervention group without mindfulness practices with the same number of sessions; measure cortisol as a physiological index of stress. Future work could also investigate whether the same protocol has the same effect in a 100% online intervention.

A further limitation is that the data were derived from couples, which means that partners’ responses may not be fully independent. Although the present analyses were appropriate for this pilot study, future research with larger samples would benefit from dyadic analytic approaches that explicitly model interdependence between partners, such as the Actor–Partner Interdependence Model.

Intra‐couple correlations at baseline were high (up to *r* = 0.91 for dyadic adjustment). Although our sensitivity analyses with random couple intercepts indicate that the main effects are robust to this dependence, individual‐level significance tests can still overstate precision. Adequately powered future trials should model interdependence confirmatorily using the Actor–Partner Interdependence Model or a two‐intercept multilevel model for distinguishable dyads (Kenny et al. 2006), and report actor and partner effects separately.

## 9 Conclusion

The results reported suggest that the effect of the combined CBCT‐mindfulness intervention was positive for both partners in terms of marital adjustment, conscious attention, and depressive symptoms, whereas no group difference was observed for marital social skills. The combination of CBT with Mindfulness may benefit partners in conflict, with communication deficits, with low income and low levels of education, even if they are beginners in meditation and therapeutic practices. This study suggests that integrated CBCT and mindfulness may be feasible and beneficial for couples from socioeconomically disadvantaged backgrounds. When supported by accessible technology (e.g., smartphone apps) and concrete skills training, such interventions may help expand access to evidence‐based strategies for promoting relational well‐being and mental health. Although the present pilot trial does not support firm conclusions about long‐term preventive effects, the findings suggest that CBCT integrated with mindfulness may be a useful approach for addressing marital conflict and may have preventive and health‐promotion potential in vulnerable populations.

## Ethics Statement

This study was approved by the National Council of Ethics in Research (CONEP) of Brazil (CAAE 09911419.1.0000.5508 /ID: 3.257.453).

## Supporting information


Supporting File

## Data Availability

The data that support the findings of this study are available from the corresponding author upon reasonable request.

## References

[jmft70169-bib-0001] Babaee, S. N. , and S. Ghahari . 2016. “Effectiveness of Communication Skills Training on Intimacy and Marital Adjustment Among Married Women.” International Journal of Medical Research & Health Sciences 5, no. 8: 375–380.

[jmft70169-bib-0002] Baer, R. A. 2003. “Mindfulness Training as a Clinical Intervention: A Conceptual and Empirical Review.” Clinical Psychology: Science and Practice 10, no. 2: 125–143. 10.1093/clipsy.bpg015.

[jmft70169-bib-0003] Barnes, S. , K. W. Brown , E. Krusemark , W. K. Campbell , and R. D. Rogge . 2007. “The Role of Mindfulness in Romantic Relationship Satisfaction and Responses to Relationship Stress.” Journal of Marital and Family Therapy 33, no. 4: 482–500. 10.1111/j.1752-0606.2007.00033.x. 17935531

[jmft70169-bib-0004] Barros, V. V. , E. H. Kozasa , I. C. W. de Souza , and T. M. Ronzani . 2015. “Validity Evidence of the Brazilian Version of the Mindful Attention Awareness Scale (MAAS).” Psicologia: Reflexão e Crítica 28, no. 1: 87–95. 10.1590/1678-7153.201528110.

[jmft70169-bib-0005] Bartholomeu, D. , C. H. S. S. Nunes , and A. A. Machado . 2008. “Traços de personalidade e habilidades sociais em universitários.” Psico‐USF 13, no. 1: 41–50. 10.1590/S1413-82712008000100006.

[jmft70169-bib-0006] Basharpoor, S. , and A. Sheykholeslami . 2015. “The Relation of Marital Adjustment and Family Functions With Quality of Life in Women.” Europe's Journal of Psychology 11, no. 3: 432–441. 10.5964/ejop.v11i3.859. PMC4873054 27247668

[jmft70169-bib-0007] Baucom, D. H. , N. B. Epstein , J. S. Kirby , and J. J. LaTaillade . 2015. “Cognitive‐Behavioral Couple Therapy.” In Clinical Handbook of Couple Therapy, 5th ed., edited by A. S. Gurman , J. L. Lebow , and D. K. Snyder , 23–60. Guilford Press.

[jmft70169-bib-0008] Beach, S. R. H. 2014. “The Couple and Family Discord Model of Depression: Updates and Future Directions.” In Interpersonal Relationships and Health: Social and Clinical Psychological Mechanisms, edited by C. R. Agnew and S. C. South , 133–155. Oxford University Press. 10.1093/acprof:oso/9780199936632.003.0007.

[jmft70169-bib-0009] Beck, A. T. 1989. Love is Never Enough: How Couples Can Overcome Misunderstandings, Resolve Conflicts, and Solve Relationship Problems Through Cognitive Therapy. HarperCollinsPublishers.

[jmft70169-bib-0010] Beck, A. T. , R. A. Steer , and G. K. Brown . 1996. Manual for the Beck Depression Inventory‐II. Psychological Corporation.

[jmft70169-bib-0011] Beck, J. S. 2011. Cognitive Behavior Therapy: Basics and Beyond, 2nd ed. Guilford Press.

[jmft70169-bib-0012] Beshai, S. 2024. “Mindfulness and CBT: A Conceptual Integration Bridging Ancient Wisdom and Modern Cognitive Theories of Psychopathology.” Frontiers in Psychology 15: 1489798. 10.3389/fpsyg.2024.1489798. 39723397 PMC11668760

[jmft70169-bib-0013] Bodenmann, G. , P. Hilpert , F. W. Nussbeck , and T. N. Bradbury . 2014. “Enhancement of Couples’ Communication and Dyadic Coping by a Self‐Directed Approach: A Randomized Controlled Trial.” Journal of Consulting and Clinical Psychology 82, no. 4: 580–591. 10.1037/a0036356. 24660673

[jmft70169-bib-0014] Bodenmann, G. , B. Plancherel , S. R. H. Beach , et al. 2008. “Effects of Coping‐Oriented Couples Therapy on Depression: A Randomized Clinical Trial.” Journal of Consulting and Clinical Psychology 76, no. 6: 944–954. 10.1037/a0013467. 19045963

[jmft70169-bib-0015] Bouchard, G. , and J. Saint‐Aubin . 2014. “Attention Deficits and Divorce.” Canadian Journal of Psychiatry 59, no. 9: 480–486. 10.1177/070674371405900904. 25565693 PMC4168810

[jmft70169-bib-0016] Brown, K. W. , and R. M. Ryan . 2003. “The Benefits of Being Present: Mindfulness and Its Role in Psychological Well‐Being.” Journal of Personality and Social Psychology 84, no. 4: 822–848. 10.1037/0022-3514.84.4.822. 12703651

[jmft70169-bib-0017] Busby, D. M. , C. Christensen , D. R. Crane , and J. H. Larson . 1995. “A Revision of the Dyadic Adjustment Scale for Use With Distressed and Nondistressed Couples: Construct Hierarchy and Multidimensional Scales.” Journal of Marital and Family Therapy 21: 289–308. 10.1111/j.1752-0606.1995.tb00163.x.

[jmft70169-bib-0018] Carson, J. W. , K. M. Carson , K. M. Gil , and D. H. Baucom . 2004. “Mindfulness‐Based Relationship Enhancement.” Behavior Therapy 35, no. 3: 471–494. 10.1016/S0005-7894(04)80028-5.

[jmft70169-bib-0019] Choi, H. , and N. F. Marks . 2013. “Marital Quality, Socioeconomic Status, and Physical Health.” Journal of Marriage and Family 75: 903–919. 10.1111/jomf.12044.

[jmft70169-bib-0020] Christensen, A. , M. Sevier , L. E. Simpson , and K. S. Gatti . 2004. “Acceptance, Mindfulness, and Change in Couple Therapy.” In Mindfulness and Acceptance Expanding the Cognitive‐Behavioral Tradition, edited by S. C. Hayes , V. M. Follette , and M. M. Linehan . Guilford Publications.

[jmft70169-bib-0021] Ciprić, A. , G. M. Hald , J. M. Strizzi , et al. 2022. “The Future of Divorce Support: Is “Digital” Enough in Presence of Conflict?” Journal of Marital and Family Therapy 48, no. 4: 1128–1146. 10.1111/jmft.12588. 35288952 PMC9790432

[jmft70169-bib-0022] Cohen, J. 1988. Statistical Power Analysis for the Behavioral Sciences. Routledge Academic. 10.4324/9780203771587.

[jmft70169-bib-0023] Cohen, S. , K. Daniel O'Leary , H. M. Foran , and S. Kliem . 2014. “Mechanisms of Change in Brief Couple Therapy for Depression.” Behavior Therapy 45, no. 3: 402–417. 10.1016/j.beth.2014.01.003. 24680234

[jmft70169-bib-0024] Cohen, S. , K. D. O'Leary , and H. Foran . 2010. “A Randomized Clinical Trial of a Brief, Problem‐Focused Couple Therapy for Depression.” Behavior Therapy 41, no. 4: 433–446. 10.1016/j.beth.2009.11.004. 21035609 PMC3536531

[jmft70169-bib-0025] Cunha, J. A. 2001. Manual da versão em português das escalas Beck. Casa do Psicólogo.

[jmft70169-bib-0026] Dancey, C. P. , and J. Reidy . 2011. Statistics Without Maths for Psychology, 5th ed. Prentice Hall.

[jmft70169-bib-0027] Dattilio, F. M. 2009. Cognitive‐Behavioral Therapy With Couples and Families. Guilford Press.

[jmft70169-bib-0028] Dattilio, F. M. , and C. A. Padesky . 1990. Cognitive Therapy With Couples. Professional Resource Exchange.

[jmft70169-bib-0029] Deci, E. L. , and R. M. Ryan . 1991. “A Motivational Approach to Self: Integration in Personality.” In Nebraska Symposium on Motivation: Perspectives on Motivation, edited by In. R. Dienstbier , 38, 237–288. University of Nebraska Press. 2130258

[jmft70169-bib-0030] Dei, D. 2019. “The Effect of Cognitive‐Behavioral Marital Therapy (CBMT) on Marital Distresses Among Married Couples in Ghana.” Texila International Journal of Psychology 4, no. 1: 31–39. 10.21522/TIJPY.2016.04.01.Art004.

[jmft70169-bib-0031] Dew, J. , S. Britt , and S. Huston . 2012. “Examining the Relationship Between Financial Issues and Divorce.” Family Relations 61, no. 4: 615–628. 10.1111/j.1741-3729.2012.00715.x.

[jmft70169-bib-0032] Departamento Intersindical de Estatística e Estudos Socioeconômicos . 2021. Pesquisa nacional da cesta básica de alimentos: Salário‐mínimo nominal e necessári. https://www.dieese.org.br/analisecestabasica/salarioMinimo.html#2022.

[jmft70169-bib-0033] Dugal, C. , G. Bakhos , C. Bélanger , and N. Godbout . 2018. “Cognitive‐Behavioral Psychotherapy for Couples: An Insight into the Treatment of Couple Hardships and Struggles.” Cognitive Behavioral Therapy and Clinical Applications 3, no. 2: 125–135. 10.5772/intechopen.72104.

[jmft70169-bib-0034] Durães, R. S. S. , T. C. Khafif , F. Lotufo‐Neto , and A. P. Serafim . 2020. “Effectiveness of Cognitive Behavioral Couple Therapy on Reducing Depression and Anxiety Symptoms and Increasing Dyadic Adjustment and Marital Social Skills: An Exploratory Study.” Family Journal 28, no. 4: 344–355. 10.1177/1066480720902410.

[jmft70169-bib-0035] Elliott, J. C. , B. A. Wallace , and B. Giesbrecht . 2014. “A Week‐Long Meditation Retreat Decouples Behavioral Measures of the Alerting and Executive Attention Networks.” Frontiers in Human Neuroscience 8, no. 69: 69. 10.3389/fnhum.2014.00069. 24596550 PMC3926190

[jmft70169-bib-0036] Epstein, N. B. , D. H. Baucom , J. S. Kirby , and J. J. LaTaillade . 2019. “Cognitive‐Behavioral Couple Therapy.” In Handbook of Cognitive‐Behavioral Therapies, edited by K. S. Dobson and D. J. A. Dozois , 433–463. The Guilford Press.

[jmft70169-bib-0037] Fischer, M. S. , D. H. Baucom , and M. J. Cohen . 2016. “Cognitive‐Behavioral Couple Therapies: Review of the Evidence for the Treatment of Relationship Distress, Psychopathology, and Chronic Health Conditions.” Family Process 55, no. 3: 423–442. 10.1111/famp.12227. 27226429

[jmft70169-bib-0038] Foale, S. , Y. Botma , and T. Heyns . 2024. “Mindfulness‐Based Interventions to Support Wellbeing of Adults in Low Socio‐Economic Settings: A Realist Review.” BMC Complementary Medicine and Therapies 24, no. 1, Article: 52. 10.1186/s12906-023-04263-7. 38267955 PMC10807132

[jmft70169-bib-0039] Germer, C. K. 2013. “Mindfulness: What is it? What Does it Matter?” In Mindfulness and Psychotherapy, 2nd ed., edited by C. K. Germer , R. D. Siegel , and P. R. Fulton . Guilford Press.

[jmft70169-bib-0040] Geschwind, N. , F. Peeters , M. Huibers , J. van Os , and M. Wichers . 2012. “Efficacy of Mindfulness‐Based Cognitive Therapy in Relation to Prior History of Depression: Randomised Controlled Trial.” British Journal of Psychiatry 201, no. 4: 320–325. 10.1192/bjp.bp.111.104851. 22878133

[jmft70169-bib-0041] Gillespie, B. , M. P. Davey , and K. Flemke . 2015. “Intimate Partners’ Perspectives on the Relational Effects of Mindfulness‐Based Stress Reduction Training: A Qualitative Research Study.” Contemporary Family Therapy 37, no. 4: 396–407. 10.1007/s10591-015-9350-x.

[jmft70169-bib-0042] Gottman, J. M. 1994. What Predicts Divorce. Erlbaum.

[jmft70169-bib-0043] Hayes‐Skelton, S. A. , and C. S. Lee . 2020. “Decentering in Mindfulness and Cognitive Restructuring for Social Anxiety: An Experimental Study of a Potential Common Mechanism.” Behavior Modification 44, no. 6: 817–840. 10.1177/0145445519850744. 31129975

[jmft70169-bib-0044] Hogendoorn, B. , M. Kalmijn , and T. Leopold . 2022. “Why Do Lower Educated People Separate More Often? Life Strains and the Gradient in Union Dissolution.” European Sociological Review 38, no. 1: 88–102. 10.1093/esr/jcab022.

[jmft70169-bib-0045] Hollist, C. S. , O. G. Falceto , L. M. Ferreira , et al. 2012. “Portuguese Translation and Validation of the Revised Dyadic Adjustment Scale.” Journal of Marital and Family Therapy 38, no. 1: 348–358. 10.1111/j.1752-0606.2012.00296.x. 22765345

[jmft70169-bib-0046] Holmboe, K. , and M. H. Johnson . 2005. “Educating Executive Attention.” Proceedings of the National Academy of Sciences of the United States of America 102, no. 41: 14479–14480. 10.1073/pnas.0507522102. 16203966 PMC1253612

[jmft70169-bib-0047] Instituto Brasileiro de Geografia e Estatística . 2024. Pesquisa Nacional por Amostra de Domicílios Contínua: *Segurança alimentar*. IBGE. https://biblioteca.ibge.gov.br/visualizacao/livros/liv102212_informativo.pdf.

[jmft70169-bib-0048] Jesus, J. G. , R. Hoffmann , and S. H. G. Miranda . 2024. “Insegurança alimentar, pobreza e distribuição de renda no Brasil.” Revista de Economia e Sociologia Rural 62, no. 4: e281936. 10.1590/1806-9479.2023.281936.

[jmft70169-bib-0049] Jha, A. P. , J. Krompinger , and M. J. Baime . 2007. “Mindfulness Training Modifies Subsystems of Attention.” Cognitive, Affective & Behavioral Neuroscience 7, no. 2: 109–119. 10.3758/cabn.7.2.109. 17672382

[jmft70169-bib-0050] Kabat‐Zinn, J. 2003. “Mindfulness‐Based Interventions in Context: Past, Present, and Future.” Clinical Psychology: Science and Practice 10, no. 2: 144–156. 10.1093/clipsy/bpg016.

[jmft70169-bib-0051] Kabat‐Zinn, J. 2013. Full Catastrophe Living: Using the Wisdom of Your Body and Mind to Face Stress, Pain, and Illness (Revised and Updated Edition). Bantam Books.

[jmft70169-bib-0052] Karney, B. R. 2021. “Socioeconomic Status and Intimate Relationships.” Annual Review of Psychology 72: 391–414. 10.1146/annurev-psych-051920-013658. PMC8179854 32886585

[jmft70169-bib-0053] Karremans, J. C. , G. Kappen , M. Schellekens , and D. Schoebi . 2020. “Comparing the Effects of a Mindfulness Versus Relaxation Intervention on Romantic Relationship Wellbeing.” Scientific Reports 10, no. 1: 21696. 10.1038/s41598-020-78919-6. 33303938 PMC7730385

[jmft70169-bib-0054] Karremans, J. C. , T. M. Pronk , and R. C. van der Wal . 2015. “Executive Control and Relationship Maintenance Processes: An Empirical Overview and Theoretical Integration.” Social and Personality Psychology Compass 9: 333–347. 10.1177/1088868315615450.

[jmft70169-bib-0055] Kenny, D. A. , D. A. Kashy , and W. L. Cook . 2006. Dyadic Data Analysis. Guilford Press.

[jmft70169-bib-0056] Kiecolt‐Glaser, J. K. 2018. “Marriage, Divorce, and the Immune System.” American Psychologist 73, no. 9: 1098–1108. 10.1037/amp0000388. 30525786 PMC6293993

[jmft70169-bib-0057] Kimmes, J. G. , M. E. Jaurequi , K. Roberts , V. W. Harris , and F. D. Fincham . 2020. “An Examination of the Association Between Relationship Mindfulness and Psychological and Relational Well‐Being in Committed Couples.” Journal of Marital and Family Therapy 46, no. 1: 30–41. 10.1111/jmft.12388. 31162689

[jmft70169-bib-0058] Kronmüller, K. T. , M. Backenstrass , D. Victor , et al. 2011. “Quality of Marital Relationship and Depression: Results of a 10‐Year Prospective Follow‐Up Study.” Journal of Affective Disorders 128, no. 1–2: 64–71. 10.1016/j.jad.2010.06.026. 20674034

[jmft70169-bib-0059] Lau, M. A. , and S. F. McMain . 2005. “Integrating Mindfulness Meditation With Cognitive and Behavioural Therapies: The Challenge of Combining Acceptance‐ and Change‐Based Strategies.” Canadian Journal of Psychiatry 50, no. 13: 863–869. 10.1177/070674370505001310. 16483122

[jmft70169-bib-0060] Le, Y. , J. Lee , D. Y. Liu , N. S. Perry , and G. K. Rhoades . 2026. “Does Sociodemographic Disadvantage Moderate the Impact of Motherwise? Findings From a Randomized Controlled Trial.” Journal of Family Psychology: JFP: Journal of the Division of Family Psychology of the American Psychological Association (Division 43) 40: 663–673. 10.1037/fam0001408. 41100291

[jmft70169-bib-0061] Leahy, R. L. 2003. Cognitive Therapy Techniques: A Practitioner's Guide. Guilford Press.

[jmft70169-bib-0062] Levy, K. N. , J. E. Beeney , R. H. Wasserman , and J. F. Clarkin . 2010. “Conflict Begets Conflict: Executive Control, Mental State Vacillations, and the Therapeutic Alliance in Treatment of Borderline Personality Disorder.” Psychotherapy Research 20, no. 4: 413–422. 10.1080/10503301003636696. 20552536

[jmft70169-bib-0063] Lin, Y. , M. E. Fisher , and J. S. Moser . 2018. “Clarifying the Relationship Between Mindfulness and Executive Attention: A Combined Behavioral and Neurophysiological Study.” Social Cognitive and Affective Neuroscience 14, no. 2: 205–215. 10.1093/scan/nsy113. PMC6374600 30535128

[jmft70169-bib-0064] Mausbach, B. T. , R. Moore , S. Roesch , V. Cardenas , and T. L. Patterson . 2010. “The Relationship Between Homework Compliance and Therapy Outcomes: An Updated Meta‐Analysis.” Cognitive Therapy and Research 34, no. 5: 429–438. 10.1007/s10608-010-9297-z. 20930925 PMC2939342

[jmft70169-bib-0065] McGill, J. , F. Adler‐Baeder , and P. Rodriguez . 2016. “Mindfully in Love: A Meta‐Analysis of the Association Between *Mindfulness* and Relationship Satisfaction.” Journal of Human Sciences and Extension 4, no. 1: 89–101. 10.54718/DDCA4089.

[jmft70169-bib-0066] Miller, R. B. , T. M. Mason , J. M. Canlas , D. Wang , D. A. Nelson , and C. H. Hart . 2013. “Marital Satisfaction and Depressive Symptoms in China.” Journal of Family Psychology 27, no. 4: 677–682. 10.1037/a0033333. 23834363

[jmft70169-bib-0067] Mosmann, C. , and D. Falcke . 2011. “Marital Conflicts: Causes and Frequency.” Revista da SPAGESP 12, no. 2: 5–16.

[jmft70169-bib-0068] Neri, M. C. 2024. “Evolução das classes econômicas brasileiras: 1976 a.” In Rio de Janeiro (2024). FGV Social.

[jmft70169-bib-0069] Neves, A. , and C. Duarte . 2015. “Depressive Symptoms, Solving Conflicts, and Marital Satisfaction in Individuals in a Relationship.” Psicologia, Saúde & Doenças 16, no. 3: 331–344.

[jmft70169-bib-0070] O'Kelly, M. , and J. Collard . 2012. “Using Mindfulness With Couples: Theory and Practice.” In Cognitive and Rational‐Emotive Behavior Therapy With Couples: Theory and Practice, edited by In. A. Vernon . Springer. 10.1007/978-1-4614-5137-2_2.

[jmft70169-bib-0071] O'Leary, K. , S. O'Neill , and S. Dockray . 2016. “A Systematic Review of the Effects of Mindfulness Interventions on Cortisol.” Journal of Health Psychology 21, no. 9: 2108–2121. 10.1177/1359105315569095. 25673371

[jmft70169-bib-0072] Perrig‐Chiello, P. , S. Hutchison , and D. Morselli . 2015. “Patterns of Psychological Adaptation to Divorce After a Long‐Term Marriage.” Journal of Social and Personal Relationships 32, no. 3: 386–405. 10.1177/0265407514533769.

[jmft70169-bib-0073] Del Prette, Z. A. P. , M. B. Villa , M. G. Freitas , and A. Del Prette . 2008. “Estabilidade temporal do Inventário de Habilidades Sociais Conjugais (IHSC).” Avaliação Psicológica 7, no. 1: 67–74.

[jmft70169-bib-0074] Qadir, F. , A. Khalid , S. Haqqani , Zill‐e‐Huma , and G. Medhin . 2013. “The Association of Marital Relationship and Perceived Social Support With Mental Health of Women in Pakistan.” BMC Public Health 13, no. 1150: 1150. 10.1186/1471-2458-13-1150. 25226599 PMC3890521

[jmft70169-bib-0075] Rector, N. A. , and S. E. Cassin . 2010. “Clinical Expertise in Cognitive Behavioural Therapy: Definition and Pathways to Acquisition.” Journal of Contemporary Psychotherapy 40: 153–161. 10.1007/s10879-010-9136-2.

[jmft70169-bib-0076] Rehman, U. S. , J. Ginting , G. Karimiha , and J. A. Goodnight . 2010. “Revisiting the Relationship Between Depressive Symptoms and Marital Communication Using an Experimental Paradigm: The Moderating Effect of Acute Sad Mood.” Behaviour Research and Therapy 48, no. 2: 97–105. 10.1016/j.brat.2009.09.013. 19878927

[jmft70169-bib-0077] Robles, T. F. 2014. “Marital Quality and Health: Implications for Marriage in the 21st Century.” Current Directions in Psychological Science 23, no. 6: 427–432. 10.1177/0963721414549043. 25544806 PMC4275835

[jmft70169-bib-0078] Robles, T. F. , R. B. Slatcher , J. M. Trombello , and M. M. McGinn . 2014. “Marital Quality and Health: A Meta‐Analytic Review.” Psychological Bulletin 140, no. 1: 140–187. 10.1037/a0031859. 23527470 PMC3872512

[jmft70169-bib-0079] Sardinha, A. , E. M. O. Falcone , and M. C. Ferreira . 2009. “The Relations Between Marital Satisfaction and Social Skills Perceived in the Spouse].” Psicologia: Teoria e Pesquisa 25, no. 3: 395–402. 10.1590/S0102-37722009000300013.

[jmft70169-bib-0080] Sbarra, D. A. , K. Hasselmo , and K. J. Bourassa . 2015. “Divorce and Health: Beyond Individual Differences.” Current Directions in Psychological Science 24, no. 2: 109–113. 10.1177/0963721414559125. 25892857 PMC4399802

[jmft70169-bib-0081] Schutte, N. S. , J. M. Malouff , C. Bobik , et al. 2001. “Emotional Intelligence and Interpersonal Relations.” Journal of Social Psychology 141, no. 4: 523–536. 10.1080/00224540109600569. 11577850

[jmft70169-bib-0082] Segal, Z. V. , and K. M. Walsh . 2016. “Mindfulness‐Based Cognitive Therapy for Residual Depressive Symptoms and Relapse Prophylaxis.” Current Opinion in Psychiatry 29, no. 1: 7–12. 10.1097/YCO.0000000000000216. 26575299 PMC4706736

[jmft70169-bib-0083] Shapiro, S. L. , L. E. Carlson , J. A. Astin , and B. Freedman . 2006. “Mechanisms of Mindfulness.” Journal of Clinical Psychology 62, no. 3: 373–386. 10.1002/jclp.20237. 16385481

[jmft70169-bib-0084] Shapiro, S. L. , G. E. Schwartz , and G. Bonner . 1998. “Effects of Mindfulness‐Based Stress Reduction on Medical and Premedical Students.” Journal of Behavioral Medicine 21, no. 6: 581–599. 10.1023/A:1018700829825. 9891256

[jmft70169-bib-0085] Shrout, M. R. , M. E. Renna , A. A. Madison , et al. 2020. “Cortisol Slopes and Conflict: A Spouse's Perceived Stress Matters.” Psychoneuroendocrinology 121, 104839: 104839. 10.1016/j.psyneuen.2020.104839. 32853875 PMC7572803

[jmft70169-bib-0086] Silva, L. P. , L. A. Vandenberghe , and M. M. P. Demarzo . 2020. “Mindfulness‐Based Interventions for the Couple Relationship: An Integrative Review].” Revista Brasileira de Terapias Cognitivas 16, no. 2: 68–74. 10.5935/1808-5687.20200011.

[jmft70169-bib-0087] Steele, L. , C. Dewa , and K. Lee . 2007. “Socioeconomic Status and Self‐Reported Barriers to Mental Health Service Use.” Canadian Journal of Psychiatry 52, no. 3: 201–206. 10.1177/070674370705200312. 17479529

[jmft70169-bib-0088] Tang, W. , and D. Kreindler . 2017. “Supporting Homework Compliance in Cognitive Behavioural Therapy: Essential Features of Mobile Apps.” JMIR Mental Health 4, no. 2: e20. 10.2196/mental.5283. 28596145 PMC5481663

[jmft70169-bib-0089] Tang, Y. Y. , B. K. Hölzel , and M. I. Posner . 2015. “The Neuroscience of Mindfulness Meditation.” Nature Reviews Neuroscience 16: 213–225. 10.1038/nrn3916. 25783612

[jmft70169-bib-0090] Tavakolizadeh, J. , M. Nejatian , and A. Soori . 2015. “The Effectiveness of Communication Skills Training on Marital Conflicts and Its Different Aspects in Women.” Procedia—Social and Behavioral Sciences 171: 214–221. 10.1016/j.sbspro.2015.01.112.

[jmft70169-bib-0091] Teasdale, J. D. , R. G. Moore , H. Hayhurst , M. Pope , S. Williams , and Z. V. Segal . 2002. “Metacognitive Awareness and Prevention of Relapse in Depression: Empirical Evidence.” Journal of Consulting and Clinical Psychology 70, no. 2: 275–287. 10.1037/0022-006X.70.2.275. 11952186

[jmft70169-bib-0092] Tian, Y. , R. Ma , N. Cui , et al. 2025. “Effects of Digital Mindfulness Training for Couples on Psychological Distress and Infant Neuropsychological Development: Randomized Controlled Trial.” Journal of Medical Internet Research 27: e77260. 10.2196/77260. 41271207 PMC12680938

[jmft70169-bib-0093] Tseng, H. W. , F. H. Chou , C. H. Chen , and Y. P. Chang . 2023. “Effects of Mindfulness‐Based Cognitive Therapy on Major Depressive Disorder With Multiple Episodes: A Systematic Review and Meta‐Analysis.” International Journal of Environmental Research and Public Health 20: 1555. 10.3390/ijerph20021555. 36674310 PMC9862388

[jmft70169-bib-0094] van der Velden, A. M. , W. Kuyken , U. Wattar , et al. 2015. “A Systematic Review of Mechanisms of Change in Mindfulness‐Based Cognitive Therapy in the Treatment of Recurrent Major Depressive Disorder.” Clinical Psychology Review 37: 26–39. 10.1016/j.cpr.2015.02.001. 25748559

[jmft70169-bib-0095] Vickers, A. J. , and D. G. Altman . 2001. “Analysing Controlled Trials With Baseline and Follow Up Measurements.” BMJ 323, no. 7321: 1123–1124. 10.1136/bmj.323.7321.1123. 11701584 PMC1121605

[jmft70169-bib-0096] Vilela, M. V. 2010. “O treino de controle do stress como tratamento do stress conjugal.” In Mecanismos neuropsicofisiológicos do stress: Teoria e aplicações clínicas, 3rd ed., edited by M. E. N. Lipp (Org.) . Casa do Psicólogo.

[jmft70169-bib-0097] Villa, M. B. , and Z. A. P. Del Prette . 2012. Inventário de Habilidades Sociais Conjugais: Manual de aplicação, apuração e interpretação. Casa do Psicólogo.

[jmft70169-bib-0098] Voldstad, A. , A. Zeas‐Sigüenza , A. Skolzkov , M. Walthaug , J. Montero‐Marín , and W. Kuyken . 2025. “The Effect of Mindfulness Interventions on Couple Relationship Satisfaction: A Systematic Review and Meta‐Analysis.” Journal of Consulting and Clinical Psychology 93, no. 6: 427–442. 10.1037/ccp0000954. 40388149

[jmft70169-bib-0099] Wachs, K. , and J. V. Cordova . 2007. “Mindful Relating: Exploring *Mindfulness* and Emotion Repertoires in Intimate Relationship.” Journal of Marital and Family Therapy 33, no. 4: 464–481. 10.1111/j.1752-0606.2007.00032.x. 17935530

[jmft70169-bib-0100] Whisman, M. A. , and R. Kaiser . 2008. “Marriage and Relationship Issues.” In Risk Factors in Depression, edited by K. S. Dobson and D. J. A. Dozois , 363–384. Elsevier Academic Press. 10.1016/B978-0-08-045078-0.00016-2.

[jmft70169-bib-0101] Williamson, H. C. , B. R. Karney , and T. N. Bradbury . 2019. “Barriers and Facilitators of Relationship Help‐Seeking Among Low‐Income Couples.” Journal of Family Psychology 33, no. 2: 234–239. 10.1037/fam0000485. 30489129 PMC6389366

[jmft70169-bib-0102] Winter, F. , A. Steffan , M. Warth , B. Ditzen , and C. Aguilar‐Raab . 2021. “Mindfulness‐Based Couple Interventions: A Systematic Literature Review.” Family Process 60, no. 3: 694–711. 10.1111/famp.12683. 34114656

[jmft70169-bib-0103] Wong, C. W. , C. S. Kwok , A. Narain , et al. 2018. “Marital Status and Risk of Cardiovascular Diseases: A Systematic Review and Meta‐Analysis.” Heart 104: 1937–1948. 10.1136/heartjnl-2018-313005. 29921571

[jmft70169-bib-0104] Zacchilli, T. L. , C. Hendrick , and S. S. Hendrick . 2009. “The Romantic Partner Conflict Scale: A New Scale to Measure Relationship Conflict.” Journal of Social and Personal Relationships 26, no. 8: 1073–1096. 10.1177/0265407509347936.

