# Supplementary material for: Effects of Cognitive Behavioral Couple Therapy With Integrated Mindfulness on Mindful Attention, Depressive Symptoms, and Dyadic Adjustment in Low‐Income Couples: A Pilot Randomized Clinical Trial
Source: J Marital Fam Ther. 2026 Aug 31;52(4):e70169. doi: 10.1111/jmft.70169 (PMC13528266; doi:10.1111/jmft.70169)
Supplement: Supplementary file 1 [file JMFT-52-0-s001.docx]

**Supplemental Material. Table S1.** Sensitivity analyses for the time × group effect (all values from the trial dataset).

| **Outcome** | **Primary mixed**  **ANOVA** | | |  | **Random couple-intercept model** | |  | **Sex-stratified**  **(time × group)** | | | |  | **Baseline-adjusted**  **group effect  (β = EG − CG)** | | | |
| --- | --- | --- | --- | --- | --- | --- | --- | --- | --- | --- | --- | --- | --- | --- | --- | --- |
|  | ***F*(2, 64)** | ***p*** | $\eta_{p}^{2}$ |  | **LRT**  **χ^2^ (2)** | ***p*** |  | **Men  *F*(2, 30)** | ***p*** | **Women  *F*(2, 30)** | ***p*** |  | **T2 β** | ***p*** | **T3 β** | ***p*** |
| **R-DAS** | 18.74 | **<.001** | .369 |  | 31.34 | **<.001** |  | 10.98 | **.0003** | 8.43 | **.0012** |  | 4.33 | .105 | 11.02 | **<.001** |
| **MAAS** | 8.09 | **<.001** | .202 |  | 15.03 | **<.001** |  | 4.25 | **.024** | 3.51 | **.043** |  | 6.79 | **.006** | 6.76 | **.004** |
| **BDI-II** | 17.00 | **<.001** | .347 |  | 28.98 | **<.001** |  | 7.75 | **.002** | 8.41 | **.001** |  | -4.28 | **.006** | -5.62 | **<.001** |
| **IHSC** | 2.05 | .138 | .060 |  | 4.21 | .122 |  | 0.46 | .634 | 2.35 | .113 |  | 3.53 | .517 | 7.47 | .137 |

*Note.* Primary mixed ANOVA = repeated-measures mixed ANOVA time × group interaction as reported in Table 3. Random couple-intercept model = linear mixed model with a random intercept for couple (participants nested within couples); the interaction was tested by a likelihood-ratio test (LRT). Sex-stratified = the same mixed ANOVA run separately for men and women. Baseline-adjusted = linear mixed model with a random couple intercept, adjusting for the baseline value of the outcome; β is the adjusted experimental − control difference at post-treatment (T2) and 3-month follow-up (T3). Bold *p* values are significant at *p* < .05. *p* values follow APA style (no leading zero).

β for mindful attention (MAAS) and marital social skills (IHSC) are expressed on the total-score scale.

R-DAS = Dyadic adjustment, MAAS = Mindful attention, IHSC = Marital social skills, BDI-II = Depressive symptoms.
